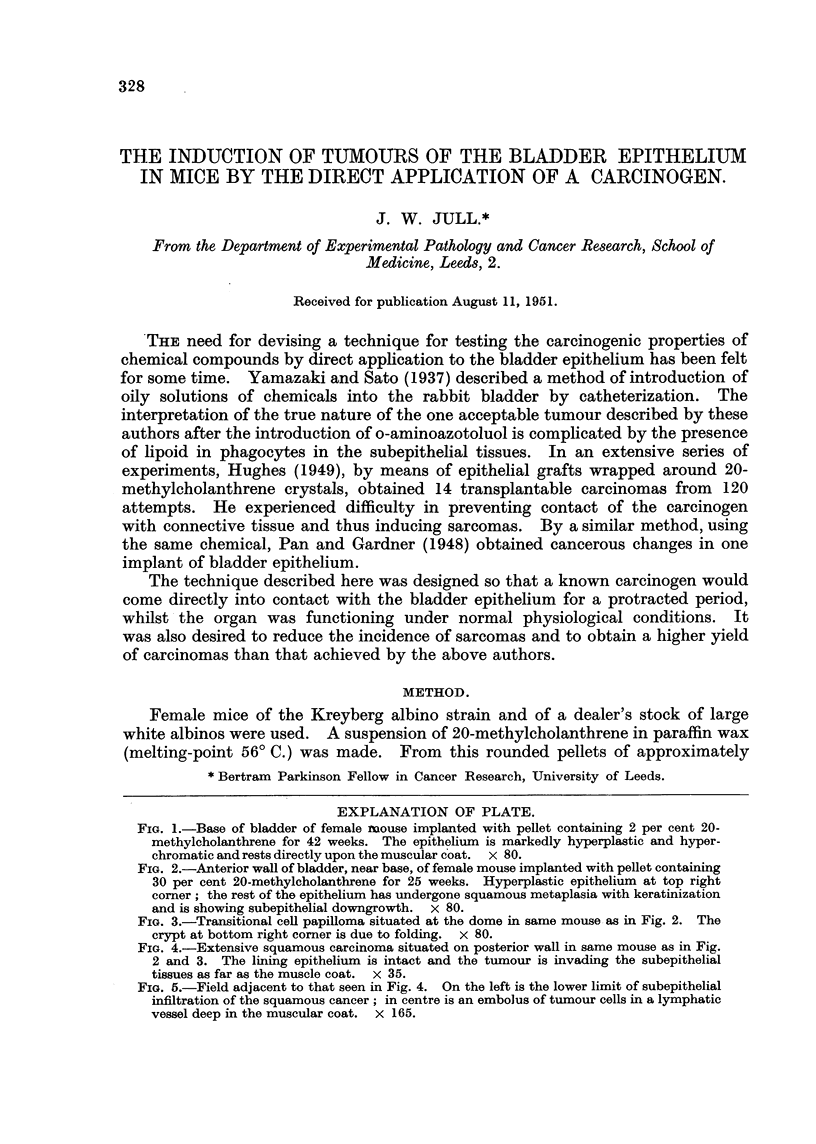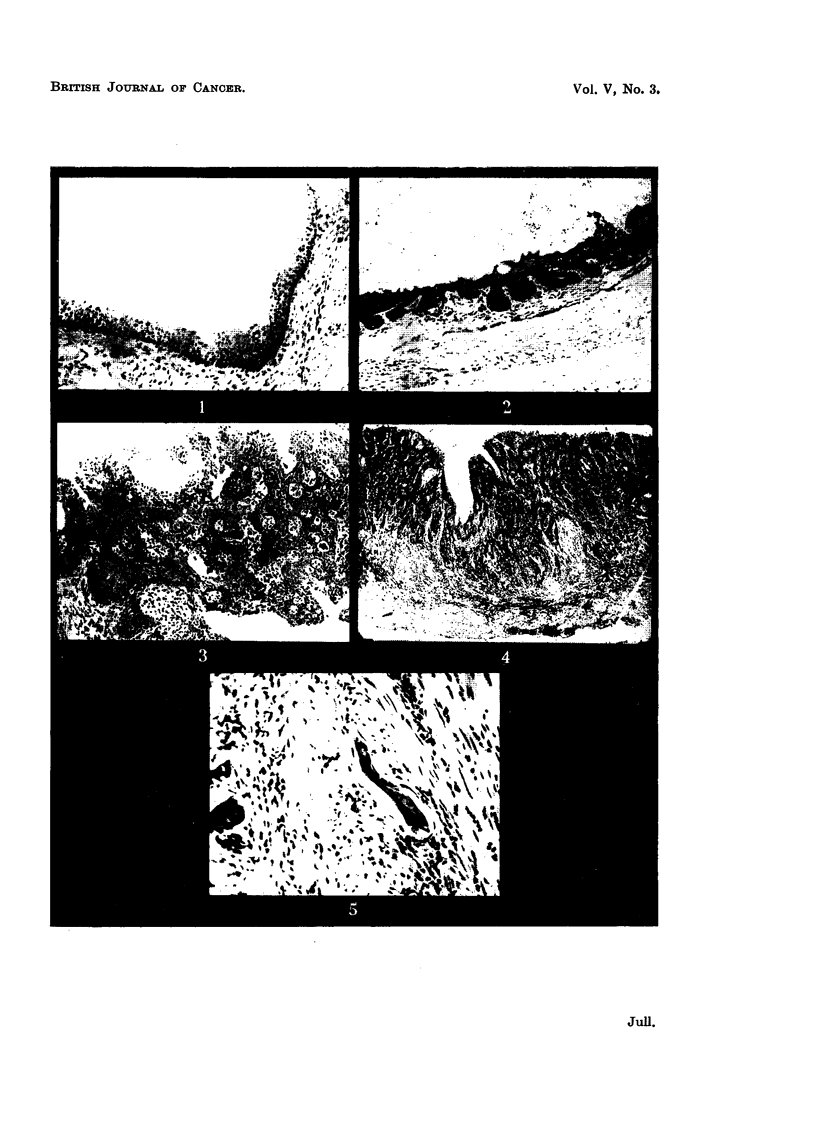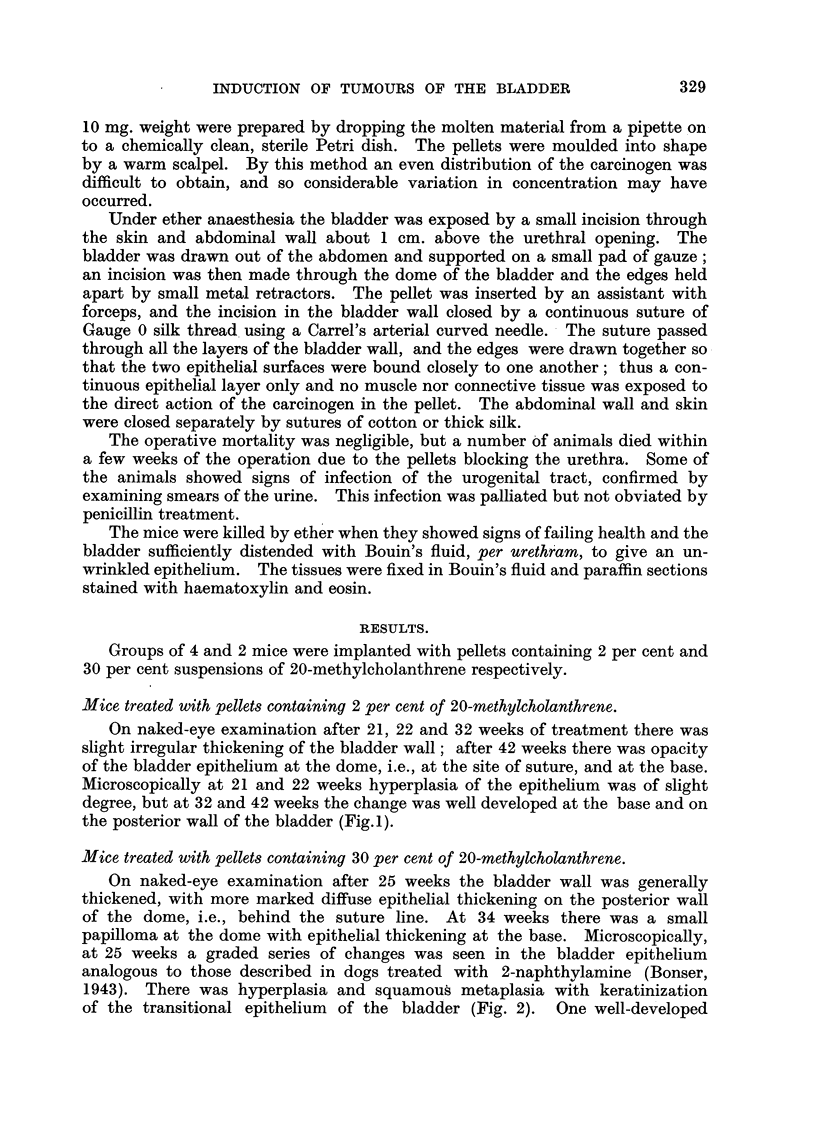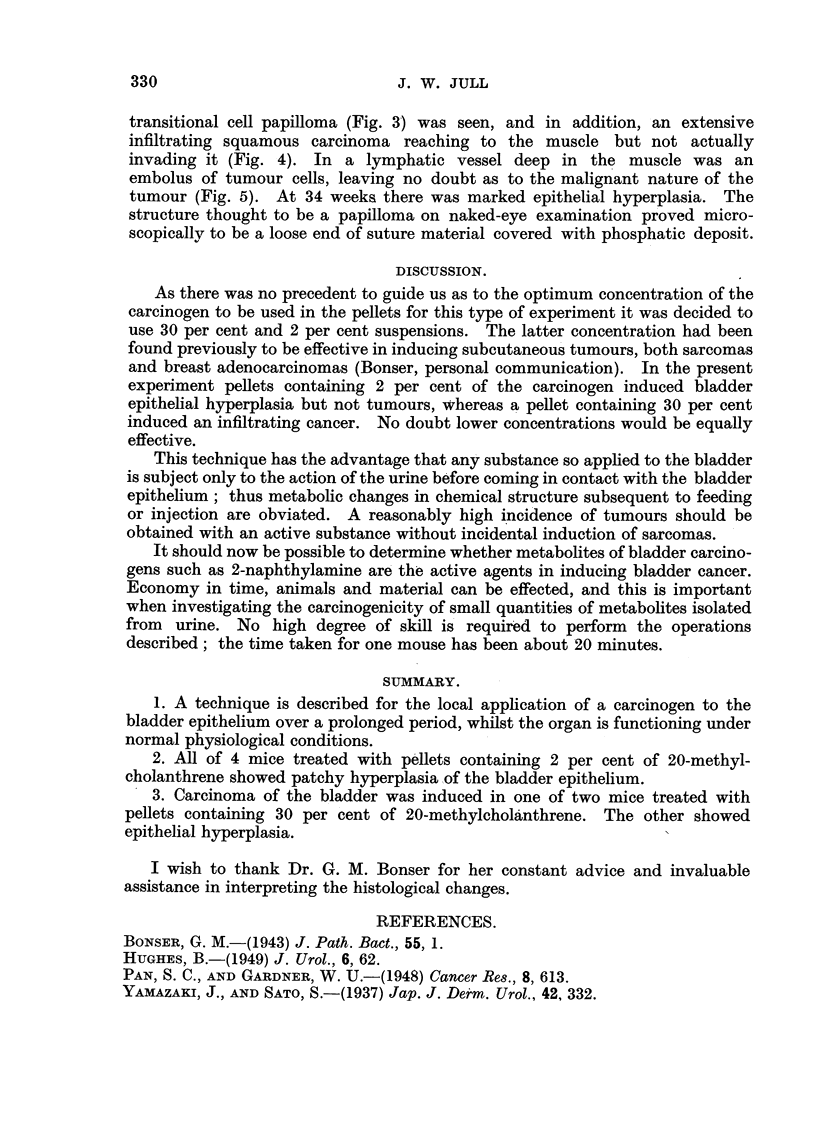# The Induction of Tumours of the Bladder Epithelium in Mice by the Direct Application of a Carcinogen

**DOI:** 10.1038/bjc.1951.34

**Published:** 1951-09

**Authors:** J. W. Jull

## Abstract

**Images:**


### THE INDUCTION OF TUMOURS OF THE BLADDER EPITHELIUM IN MICE BY THE DIRECT APPLICATION OF A CARCINOGEN.

J. W. JULL.*

*From the Department of Experimental Pathology and Cancer Research, School of Medicine, Leeds, 2.*

Received for publication August 11, 1951.

THE need for devising a technique for testing the carcinogenic properties of chemical compounds by direct application to the bladder epithelium has been felt for some time. Yamazaki and Sato (1937) described a method of introduction of oily solutions of chemicals into the rabbit bladder by catheterization. The interpretation of the true nature of the one acceptable tumour described by these authors after the introduction of o-aminoazotoluol is complicated by the presence of lipoid in phagocytes in the subepithelial tissues. In an extensive series of experiments, Hughes (1949), by means of epithelial grafts wrapped around 20-methylcholanthrene crystals, obtained 14 transplantable carcinomas from 120 attempts. He experienced difficulty in preventing contact of the carcinogen with connective tissue and thus inducing sarcomas. By a similar method, using the same chemical, Pan and Gardner (1948) obtained cancerous changes in one implant of bladder epithelium.

The technique described here was designed so that a known carcinogen would come directly into contact with the bladder epithelium for a protracted period, whilst the organ was functioning under normal physiological conditions. It was also desired to reduce the incidence of sarcomas and to obtain a higher yield of carcinomas than that achieved by the above authors.

### METHOD.

Female mice of the Kreyberg albino strain and of a dealer's stock of large white albinos were used. A suspension of 20-methylcholanthrene in paraffin wax (melting-point 56° C.) was made. From this rounded pellets of approximately

* Bertram Parkinson Fellow in Cancer Research, University of Leeds.

### EXPLANATION OF PLATE.

FIG. 1.—Base of bladder of female mouse implanted with pellet containing 2 per cent 20-methylcholanthrene for 42 weeks. The epithelium is markedly hyperplastic and hyperchromatic and rests directly upon the muscular coat. × 80.

FIG. 2.—Anterior wall of bladder, near base, of female mouse implanted with pellet containing 30 per cent 20-methylcholanthrene for 25 weeks. Hyperplastic epithelium at top right corner ; the rest of the epithelium has undergone squamous metaplasia with keratinization and is showing subepithelial downgrowth. × 80.

FIG. 3.—Transitional cell papilloma situated at the dome in same mouse as in Fig. 2. The crypt at bottom right corner is due to folding. × 80.

FIG. 4.—Extensive squamous carcinoma situated on posterior wall in same mouse as in Fig. 2 and 3. The lining epithelium is intact and the tumour is invading the subepithelial tissues as far as the muscle coat. × 35.

FIG. 5.—Field adjacent to that seen in Fig. 4. On the left is the lower limit of subepithelial infiltration of the squamous cancer ; in centre is an embolus of tumour cells in a lymphatic vessel deep in the muscular coat. × 165.

Jull.

10 mg. weight were prepared by dropping the molten material from a pipette on to a chemically clean, sterile Petri dish. The pellets were moulded into shape by a warm scalpel. By this method an even distribution of the carcinogen was difficult to obtain, and so considerable variation in concentration may have occurred.

Under ether anaesthesia the bladder was exposed by a small incision through the skin and abdominal wall about 1 cm. above the urethral opening. The bladder was drawn out of the abdomen and supported on a small pad of gauze; an incision was then made through the dome of the bladder and the edges held apart by small metal retractors. The pellet was inserted by an assistant with forceps, and the incision in the bladder wall closed by a continuous suture of Gauge 0 silk thread using a Carrel's arterial curved needle. The suture passed through all the layers of the bladder wall, and the edges were drawn together so that the two epithelial surfaces were bound closely to one another; thus a continuous epithelial layer only and no muscle nor connective tissue was exposed to the direct action of the carcinogen in the pellet. The abdominal wall and skin were closed separately by sutures of cotton or thick silk.

The operative mortality was negligible, but a number of animals died within a few weeks of the operation due to the pellets blocking the urethra. Some of the animals showed signs of infection of the urogenital tract, confirmed by examining smears of the urine. This infection was palliated but not obviated by penicillin treatment.

The mice were killed by ether when they showed signs of failing health and the bladder sufficiently distended with Bouin's fluid, *per urethram*, to give an unwrinkled epithelium. The tissues were fixed in Bouin's fluid and paraffin sections stained with haematoxylin and eosin.

### RESULTS.

Groups of 4 and 2 mice were implanted with pellets containing 2 per cent and 30 per cent suspensions of 20-methylcholanthrene respectively.

*Mice treated with pellets containing* 2 *per cent of* 20-*methylcholanthrene.*

On naked-eye examination after 21, 22 and 32 weeks of treatment there was slight irregular thickening of the bladder wall; after 42 weeks there was opacity of the bladder epithelium at the dome, i.e., at the site of suture, and at the base. Microscopically at 21 and 22 weeks hyperplasia of the epithelium was of slight degree, but at 32 and 42 weeks the change was well developed at the base and on the posterior wall of the bladder (Fig.1).

*Mice treated with pellets containing* 30 *per cent of* 20-*methylcholanthrene.*

On naked-eye examination after 25 weeks the bladder wall was generally thickened, with more marked diffuse epithelial thickening on the posterior wall of the dome, i.e., behind the suture line. At 34 weeks there was a small papilloma at the dome with epithelial thickening at the base. Microscopically, at 25 weeks a graded series of changes was seen in the bladder epithelium analogous to those described in dogs treated with 2-naphthylamine (Bonser, 1943). There was hyperplasia and squamous metaplasia with keratinization of the transitional epithelium of the bladder (Fig. 2). One well-developed transitional cell papilloma (Fig. 3) was seen, and in addition, an extensive infiltrating squamous carcinoma reaching to the muscle but not actually invading it (Fig. 4). In a lymphatic vessel deep in the muscle was an embolus of tumour cells, leaving no doubt as to the malignant nature of the tumour (Fig. 5). At 34 weeks there was marked epithelial hyperplasia. The structure thought to be a papilloma on naked-eye examination proved microscopically to be a loose end of suture material covered with phosphatic deposit.

### DISCUSSION.

As there was no precedent to guide us as to the optimum concentration of the carcinogen to be used in the pellets for this type of experiment it was decided to use 30 per cent and 2 per cent suspensions. The latter concentration had been found previously to be effective in inducing subcutaneous tumours, both sarcomas and breast adenocarcinomas (Bonser, personal communication). In the present experiment pellets containing 2 per cent of the carcinogen induced bladder epithelial hyperplasia but not tumours, whereas a pellet containing 30 per cent induced an infiltrating cancer. No doubt lower concentrations would be equally effective.

This technique has the advantage that any substance so applied to the bladder is subject only to the action of the urine before coming in contact with the bladder epithelium ; thus metabolic changes in chemical structure subsequent to feeding or injection are obviated. A reasonably high incidence of tumours should be obtained with an active substance without incidental induction of sarcomas.

It should now be possible to determine whether metabolites of bladder carcinogens such as 2-naphthylamine are the active agents in inducing bladder cancer. Economy in time, animals and material can be effected, and this is important when investigating the carcinogenicity of small quantities of metabolites isolated from urine. No high degree of skill is required to perform the operations described ; the time taken for one mouse has been about 20 minutes.

### SUMMARY.

1. A technique is described for the local application of a carcinogen to the bladder epithelium over a prolonged period, whilst the organ is functioning under normal physiological conditions.

2. All of 4 mice treated with pellets containing 2 per cent of 20-methylcholanthrene showed patchy hyperplasia of the bladder epithelium.

3. Carcinoma of the bladder was induced in one of two mice treated with pellets containing 30 per cent of 20-methylcholanthrene. The other showed epithelial hyperplasia.

I wish to thank Dr. G. M. Bonser for her constant advice and invaluable assistance in interpreting the histological changes.

### REFERENCES.

Bonser, G. M.—(1943) *J. Path. Bact.*, **55**, 1.
Hughes, B.—(1949) *J. Urol.*, **6**, 62.
Pan, S. C., and Gardner, W. U.—(1948) *Cancer Res.*, **8**, 613.
Yamazaki, J., and Sato, S.—(1937) *Jap. J. Derm. Urol.*, **42**, 332.